# Punishment in Poor-Law Infirmaries

**Published:** 1913-11-08

**Authors:** 


### PUNISHMENT IN POOR-LAW INFIRMARIES.

Publicity has recently been given to the question of the propriety of the punishment of children in rate-supported hospitals, and the usual acute differences of opinion have arisen. The powers of the medical superintendent under the Local Government Board are therefore worth notice.

The infirmaries, or Poor-Law hospitals, remain an integral part of the Poor-Law administration, and their management is subject to regulations, modified to meet their special needs, founded upon the Poor-Law Orders of the central governing authority, the Local Government Board. The scheme for the maintenance of order and decorum in workhouses is founded upon the General Consolidated Order of 1847, in which are detailed at considerable length the forms of misbehaviour which constitute disorderly or refractory conduct. Punishment varies from that of a charge before the Justices of the Peace (in the more serious cases) to the temporary substitution of bread and water for the ordinary diet, confinement in a separate room, or restriction of leave of absence; in some cases a certificate from the medical officer is requisite before the diet may be modified. The corporal punishment of adults was prohibited in the reign of George III.

In the case of children it is remarked in a footnote to Articles 136-142 that "the expediency of" corporal punishment is a "difficult subject, and all classes of society are somewhat divided in opinion respecting it." It is laid down that no corporal punishment shall be inflicted on any female child, nor on any male child, until two hours shall have elapsed from the commission of the offence for which such punishment is inflicted, and then only with an approved "rod or other instrument," and by the schoolmaster or master, who shall both, if possible, be present. No child under twelve years of age is to be punished by confinement in a dark room, or during the night. All cases of punishment, both of adults and children, must be entered in a book, which is produced to the Guardians.

The medical superintendent of an infirmary is the master of that, in the larger cases now separated, part of the workhouse and has powers similar to those of the master in the workhouse. He has not the power, usually possessed by a medical officer of a "voluntary" hospital, to discharge or to advise the discharge of a patient. He has usually authority to transfer any patient, whose condition permits, to the workhouse; to this course, however, as to that of taking the patient before a magistrate, there may be objections, the offender being a sick person. Restriction of diet may be legally ordered, but, in this again, the medical superintendent, who has ordered a certain diet as being suited to the patient's condition of health, is placed in a somewhat false position when, like Pooh Bah in "The Mikado," he as a disciplinarian orders something different. He may adopt such minor restrictions as confining a patient to bed or removing him to another ward, though only under very exceptional circumstances should the removal be to a ward containing patients insane or otherwise objectionable. A medical man may become legally responsible should he endanger any patient's life, person, or health. With regard to children the medical superintendent has undoubtedly the powers above referred to, and, in the event of his deeming the corporal punishment of male children to be necessary, is legally justified in accepting the duty, as "master," meting out that punishment himself. It is absolutely improper for a nurse in charge of sick children to use any form of corporal punishment. In the mind of the uninitiated there at once arises the question: Can children so ill as to be kept in a hospital ward need corporal punishment? Undoubtedly they may be naughty as well in a sick ward as elsewhere. The same is true of convalescent children.

The fact that means of punishment are so few shows that such means have proved unnecessary, and is a tribute to the conduct of both staff and patients. The infirmary medical superintendent treads a path beset with almost as many difficulties as that of a Cabinet Minister. He must wield the powers of his limited autocracy with infinite tact. Without contravening any Order or exceeding the limits of those powers, whilst carrying out what he deems to be strictly his duty, he may easily forget the apostolic view that many things are lawful which are not expedient and may lay himself open to criticism. In such a matter as the chastisement of naughty boys his powers and duties appear to be clearly defined. At the moment the powers of an infirmary medical superintendent are all with which we are concerned. The ethics of punishment is a wider and much more controversial matter.

